# Myricetin activates the Caspase-3/GSDME pathway *via* ER stress induction of pyroptosis in lung cancer cells

**DOI:** 10.3389/fphar.2022.959938

**Published:** 2022-08-26

**Authors:** Jicheng Han, Cheng Cheng, Jinxin Zhang, Jinbo Fang, Wei Yao, Yilong Zhu, Zhiru Xiu, Ningyi Jin, Huijun Lu, Xiao Li, Yiquan Li

**Affiliations:** ^1^ Academician Workstation, Changchun University of Chinese Medicine, Changchun, China; ^2^ Changchun Veterinary Research Institute, Chinese Academy of Agricultural Sciences, Changchun, China; ^3^ College of Life Science and Technology, Changchun University of Science and Technology, Changchun, China; ^4^ Healthcare Department, Agency for Offices Administration, Beijing, China

**Keywords:** myricetin, GSDME, er stress, ROS, pyroptosis

## Abstract

Pyroptosis is related to the occurrence, development, and therapeutic response of tumors, mediated by the proteins of the Gasdermin family. These proteins have become potential biomarkers for cancer treatment, and their agonists are likely to become a new direction in research and development of antitumor drugs. In this study, we found that myricetin has an inhibitory effect on lung cancer cells of the activation of pyroptosis. Analysis of the expression of Gasdermin family proteins revealed that this phenomenon was caused by the cleavage of GSDME. Subsequently, specific inhibitors, we found that caspase-3 was its upstream activation factor. In addition, mitochondrial and endoplasmic reticulum (ER) analysis showed that myricetin can cause endoplasmic reticulum stress and increase reactive oxygen species (ROS) levels. Subsequent inhibition of caspase-12 revealed that the expression levels of cleaved-caspase-3 and cleaved-GSDME were significantly reduced, resulting in the inhibition of pyroptosis. Using *in vivo* experiments, we also found that the treatment with myricetin can reduce tumor volume and significantly increase the level of pyroptosis-related proteins in tumor tissues. Overall, our findings show that myricetin induces cell death of lung cancer cells primarily through an ER stress pathway-induced pyroptosis. Therefore, myricetin has the potential to be used as a pyroptosis agonist in research and development of antitumor drugs.

## Introduction

Lung cancer is a common solid cancer, that has the highest cancer-related mortality in the world (Huang et al., 2019), accounting for 1.8 million deaths per year (Torre et al., 2015). Among all cancers, lung cancer has the worst prognosis (Shin et al., 2019). At the time of detection, approximately 70% of lung cancer patients have other metastatic diseases. Based on histopathological features, lung cancer is mainly divided into non-small cell lung cancer (NSCLC) and small cell lung cancer (SCLC). NSCLC accounts for 80–85% of the total number of lung cancers and the 5-year survival rate of patients with early-stage NSCLC can reach 50–60%, while the 5-year survival rate of patients with advanced NSCLC is generally only about 10%. SCLC accounts for 15–20% of all lung cancers (Osmani et al., 2018) and is one of the most challenging cancers with a 5-year survival rate of 4–5% (Harris et al., 2012). SCLC is an aggressive malignancy that is most seen in patients who have smoked or are smokers. However, SCLC can occasionally be diagnosed in individuals who have never smoked or mildly smoked (Ou et al., 2009; Varghese et al., 2014; Ogino et al., 2021). Routine treatments for patients with lung cancer mainly include chemotherapy and radiotherapy, while surgical resection is rarely performed for patients with advanced lung cancer (Zheng, 2016; Stinchcombe, 2017; Yang et al., 2019). At present, the most common drugs for the treatment of lung cancer are irinotecan, topotecan, etoposide, gemcitabine, and paclitaxel. However, lung cancer is sensitive to chemotherapeutic drugs, which may lead to a general decline in patients’ quality of life (Gu et al., 2009; Reddy et al., 2020). Therefore, developing more effective therapeutic drugs has become a hot topic in this field.

Cell pyroptosis is a newly discovered type of programmed cell death (PCD) characterized by a continuous expansion of cells that leads to a rupture cell membranes (Galluzzi et al., 2018). Cell pyroptosis was originally thought to be an innate immune mechanism, mediated by pro-inflammatory caspases to promote the cellular pyroptosis of immune cells, such as macrophages, monocytes, dendritic cells (DC), and T cells. This event was also thought to be stimulated pathogens or their products (Schroder and Tschopp, 2010; Jorgensen et al., 2017). With the discovery of the gasdermin family of proteins, the research scope of cell pyroptosis was expanded. Recent studies showed that cancer cells can also mediate cell pyroptosis through caspases (Yu et al., 2021). The molecular mechanisms of cell pyroptosis include the classical pathway (caspase-1), the non-classical pathway (caspase-4/5/11), the caspase-3 pathway, and the granzyme-mediated pathway (Yu et al., 2021). Studies have found that cell pyroptosis is closely related to the development and occurrence of melanoma, breast cancer, colorectal cancer, gastric cancer, hepatocellular carcinoma, lung cancer, cervical cancer, and leukemia (Yu et al., 2021).

GSDME belongs to the gasdermin (GSDM) family and was originally identified as DFNA5 (deafness, autosomal dominant 5) (Yu et al., 2021). Currently, the gasdermin (GSDM) family includes six GSDM genes: GSDMA, GSDMB, GSDMC, GSDMD, GSDME, and Pejvakin (PJVK) (Tamura et al., 2007). All conserved N-terminal domains of the gasdermin (GSDM) family members, with the exception of PJVK, were shown to be involved in the execution of necrotic cell death (Ding et al., 2016). A study found that activated caspase-3 can cleave DFNA5/GSDME after its successful induction of apoptosis, resulting in the production of a GSDME-N fragment that penetrates cell membranes to induce pyroptosis (Rogers et al., 2017). Other researchers confirmed the cleavage and activation of GSDME by caspase 3. The authors also found that the expression of wild-type DFNA5/GSDME in tumor cells reverses caspase-3 activation, leading to a shift from apoptosis to pyroptosis. After chemotherapeutic treatment, caspase-3-induced cleavage of GSDME determines cellular pyroptosis in some GSDME-expressing cancer cells (Wang et al., 2017). GSDME is silenced in most cancer cells but is expressed in many normal tissues. Currently, many studies have pointed out to the potential therapeutic use of GSDME as a new research direction in the development of cancer chemotherapeutic drugs (Wang et al., 2017).

In this study, we selected the natural product myricetin to explore its inhibitory effect on lung cancer cells and clarify its inhibitory mechanism on lung cancer cells. This approach will provide a scientific basis for the development of lung cancer therapeutic drugs.

## Materials and methods

### Cells and animals

The human lung cancer cell lines NCI-H446, A549 and mouse lung cancer Lewis cells were cultured in RPMI-1640 medium containing 10% FBS and double antibodies. The human normal lung epithelial cells BEAS-2B were cultured in DMEM medium containing 10% FBS and double antibodies. All cell lines were obtained from Wuhan Punosei Life Technology Co., Ltd. The 5-week-old BALB/c Nude mice that were used in this study were purchased from Beijing Viton Lihua Laboratory Animal Technology Co., Ltd.

### Western blot

NCI-H446 cells, A549, and Lewis cells were treated with different concentrations of myricetin. The myricetin + inhibitor treatment group and the control group were concomitantly set up in parts of study. The caspase-3 inhibitor (Cat#556547, AAT Bioquest) and caspase-12 inhibitor (Cat#ab141383, Abcam) were used in this study. After 48 h of culture, the cells were collected and centrifuged to remove the supernatants, and the cells’ total proteins were extracted by a protein extraction kit (Cat#DE101-01, TransGen Biotech). After protein quantification, 30 μg total protein was added to SDS-PAGE for separation that was followed by transfer onto a PVDF membrane. After incubation for 2 h with a blocking solution, the corresponding primary antibody was added, and the membrane was incubated overnight at 4°C. After three times washing with TBST, the membrane was incubated with a goat anti-rabbit or goat anti-mouse secondary antibody for 40 min. Finally, the proteins were detected by enhanced chemiluminescence (ECL). The (anti-GSDMD (Cat#ab209845) and anti-GSDME (Cat#ab215191) antibodies were purchased from Abcam. The anti-GAPDH (Cat#5174), anti-cleaved-caspase-3 (Cat#9664), anti-caspase-3 (Cat#9662), anti-caspase-4(Cat#4450), anti-caspase-5(Cat#46680), anti-caspase-6 (Cat#9762), anti-cleaved- cleaved-caspase-6 (Cat#9761), anti-cleaved-caspase-7 (Cat#8438), anti-caspase-7 (Cat#12827), anti-caspase-8 (Cat#4790), antibodies were purchased from Cell Signaling Technology.

### Crystal violet assay

NCI-H446 and A549 cells were passaged in 6-well plates (4×10^5^ cells/well), incubated at 37°C in a 5% CO_2_ cell incubator for 12h, and treated with different concentrations of myricetin (Cat#HY-15097, MedChemExpress). Meanwhile, a control group was set up. After 48 h of culture, the culture media were discarded, and the wells were washed three times with PBS. An amount of 600 μL 0.4% crystal violet (Cat#C0121, Beyotime Biotechnology) staining solution was added to each well and the staining solution was discarded after 10 min staining at room temperature. The wells were rinsed three times with PBS and conserved in a dry and cool environment for photographing and analysis.

### Cell migration and invasion assay

NCI-H446 and A549 cells were passaged in 6-well plates (1 × 10^6^ cells/well) and incubated at 37°C in a 5% CO_2_ cell incubator for 12 h. After incubation, the cells in each well were scratched with a micropipette tip and photographed. Subsequently, myricetin with different concentrations was added, and the control group was set. After 48 h of culture, the scratches were photographed.

NCI-H446 and A549 cells were passaged in 24-well plates (5 × 10^4^ cells/well), incubated at 37°C in a 5% CO_2_ cell incubator for 12 h, and then treated with different concentrations of myricetin. Meanwhile, a control group was set up. After 48 h of culture, the cells were collected, incubated in a Transwell chamber (Cat#3460, CORNING) (matrix gel was added in advance in the chamber) for 24 h, and then the chamber was fixed in methanol for 30 min. After crystal violet staining, the Transwell chamber was observed under a microscope.

### Microscope and electron microscope observation

NCI-H446 and A549 cells were passaged in 6-well plates (4 × 10^5^ cells/well), and myricetin was added after 12 h culture. Meanwhile, a Mock control group was set up. After 48 h of culture, some cells were observed under a microscope, while others were centrifuged and fixed with electron microscope fixative solution at 4°C for 24 h before electron microscopy observation.

### Flow cytometry

For flow cytometry analysis, reference was made to previous studies for the detection of apoptotic levels in NCI-H446 and A549 cells (Li T. et al., 2022). NCI-H446 and A549 cells were passaged in 12-well plates (2 × 10^5^ cells/well), incubated at 37°C in a 5% CO_2_ cell incubator for 12 h, and then treated with different concentrations of myricetin. Meanwhile, a control group was set up. After 48 h of culture, the cells from each group were collected and stained with Annexin V-FITC/PI detection kit (Cat#556547, BD), washed three times with PBS, and detected by flow cytometry according to the kit instructions.

### LDH release assay

In this study, the LDH index of cell pyroptosis was detected by Cytotox 96 Non-Radioactive Cytotoxity Assay Kit (Cat#G1780, Promega). The experimental procedures were performed according to the instruction manual. After detection, the absorbance was measured at 450 nm using a microplate reader.

### ROS detection assay

The cells were seeded into a 12-well plate (2 × 10^5^ cells/well) and cultured for 24 h. Then, the cells were treated with different concentrations of myricetin. After 48 h, the cells were collected, and then 5 μM DCFH-DA dye solution (Cat#D6883, Sigma) was added. The cells were stained for 30 min in the dark. Flow cytometry was performed to observe the changes in ROS levels.

### Mouse tumor model assay

After 5–7 days of adaptive hosting of SPF grade 5-week-old BALB/c nude mice, 100 μL cell suspension containing NCI-H446 and A549 cells (2 × 10^7^ cells/mL) were subcutaneously injected into the right thigh of each BALB/c nude mouse. Seven days after inoculation, the nude mice were randomly divided into two groups. One group was given myricetin (10 mg/kg) every 2 days by intratumoral administration, and the control group was given the same volume of PBS. The nude mice in each group were weighed once a week. On day 21, they were sacrificed, and the tumors’ volume was measured. Some tumors were separately analyzed by immunohistochemistry and WB analysis.

### Statistical analysis

All experiments were independently conducted at least three times, and the results were expressed as mean ± standard deviation (SD). The GraphPadPrism8.0 software was used for statistical analysis or for the analysis of variance (ANOVA) of the unpaired double-tailed students-test. Significance levels were defined as *p < 0.05, **p < 0.01, ***p < 0.001, and ****p < 0.0001.

## Results

### Myricetin reduces the viability of lung cancer cells

**FIGURE 1:**
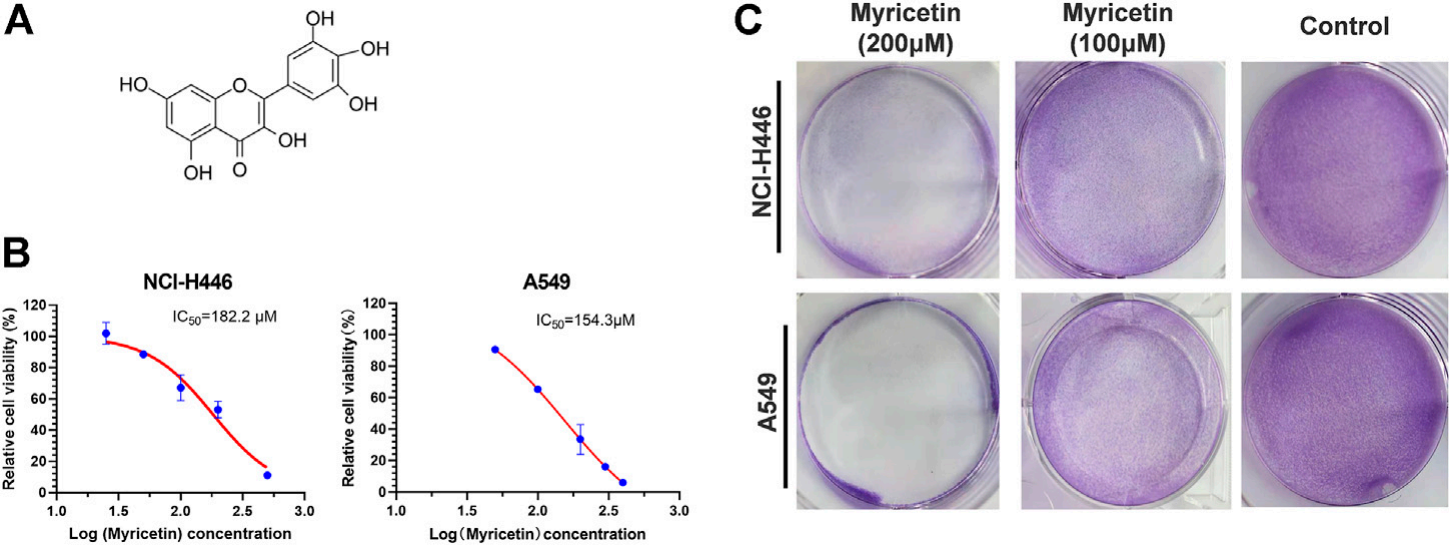
Inhibitory effect of myricetin on lung cancer cells. **(A)** Chemical Structure of Myricetin. **(B)**Half maximal inhibitory concentration (IC50) of Myricetin inhibiting the proliferation of NCI-H446 and A549 cells. **(C)** Crystal violet staining of NCI-H446 and A549 cells treated with Myricetin. The results were presented as mean ± standard deviation.

Different concentrations of myricetin (Figure 1A) were added into 96-well plates to treat NCI-H446 and A549 cells for 48 h and then the CCK-8 reagent was added for detection by a microplate reader. The used IC50 of myricetin were 182.2 ± 5.2 for NCI-H446 and 154.3 ± 4.6 for A549 (Figure 1B). The same treatment was used for BEAS-2B with an IC50 of 634.1 ± 6.5 (Supplementary Figure S1A). NCI-H446 and A549 were seeded into a 6-well plate for 12 h, and after treatment of the cells with myricetin for 24 h, the plate was taken out for crystal violet staining. The results showed that the number of myricetin-treated cells was significantly reduced (Figure 1C). Taken together, the results above indicate that myricetin can effectively induce cell death of lung cancer cells.

### Myricetin inhibits the migration and invasion of lung cancer cells

**FIGURE 2:**
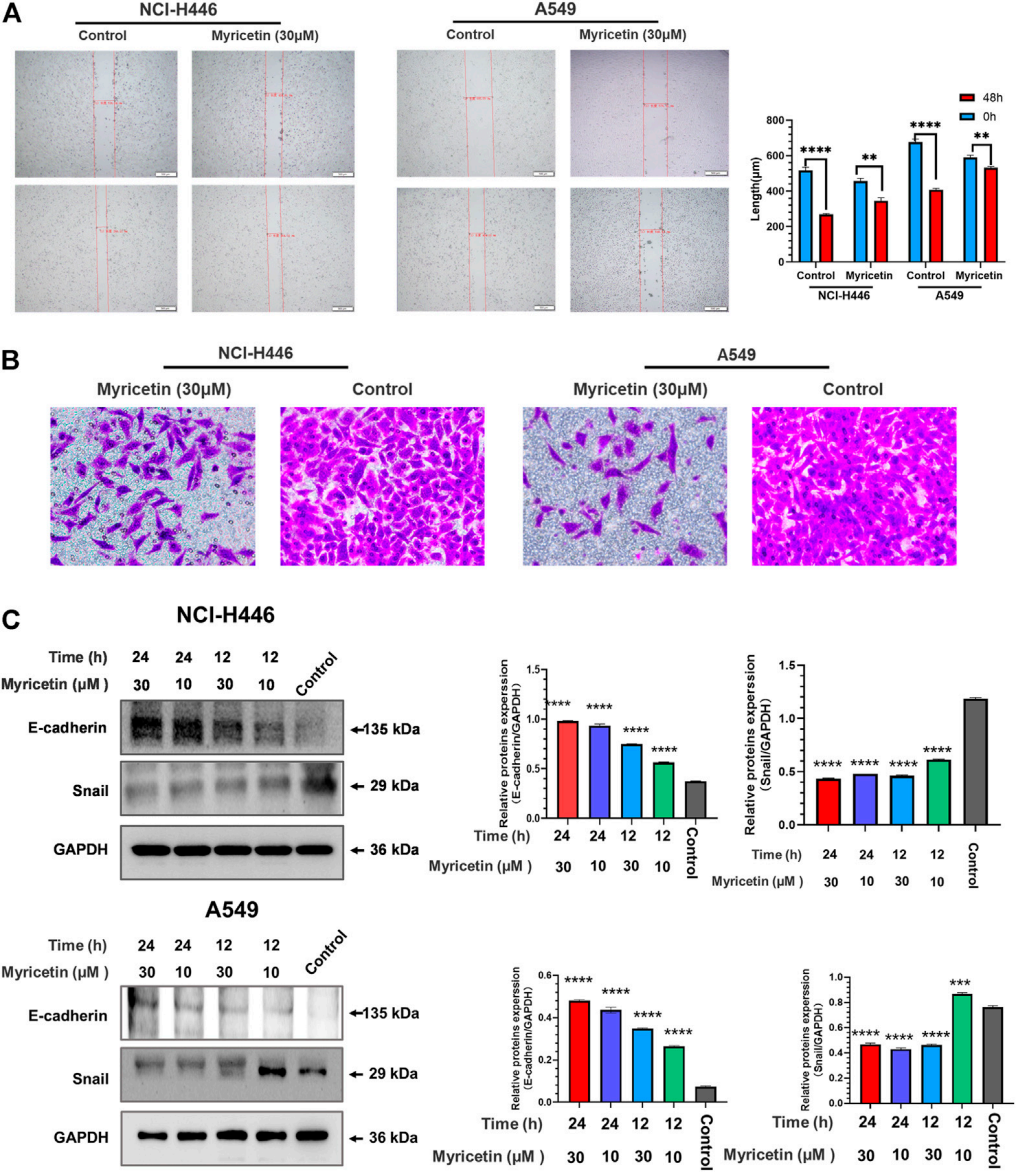
Analysis of myricetin inhibition of lung cancer cells’ migration and invasion. Wound scratch assay of NCI-H446 and A549 cells treated with myricetin (30 μM) and its statistical analysis **(A)**. **(B)**Migration analysis of myricetin (30 μM) -treated NCI-H446 and A549 cells. Western blot analyses of E-cadherin and Snail expression in NCI-H446 and A549 cells after myricetin (10 and 30 μM) treatment and their corresponding statistical analyses **(C)**. The results were presented as mean ± standard deviation (****p < 0.0001, when compared with control).

NCI-H446 and A549 cells were plated into 6-well plates for 12 h before scratching experiments. The results of the scratch test are shown in Figure 2A. Myricetin was able to significantly inhibit cell migration. As shown in Figure 2B, myricetin effectively inhibited the invasion of cells from the upper chamber to the lower chamber, and myricetin’s ability to inhibit the invasion of cells were significantly higher than that in the control group. The protein lysates from myricetin-treated NCI-H446 and A549 cells (at different concentrations) were collected for protein analysis by western blot.

The results showed that treatments of cells with 10 and 30 μM myricetin result in a significant increase in E-cadherin protein and a significant decrease in Snail protein (Figure 2C). These results demonstrate that myricetin can effectively inhibit the migration and invasion of lung cancer cells.

### Myricetin induces pyroptosis by GSDME

**FIGURE 3:**
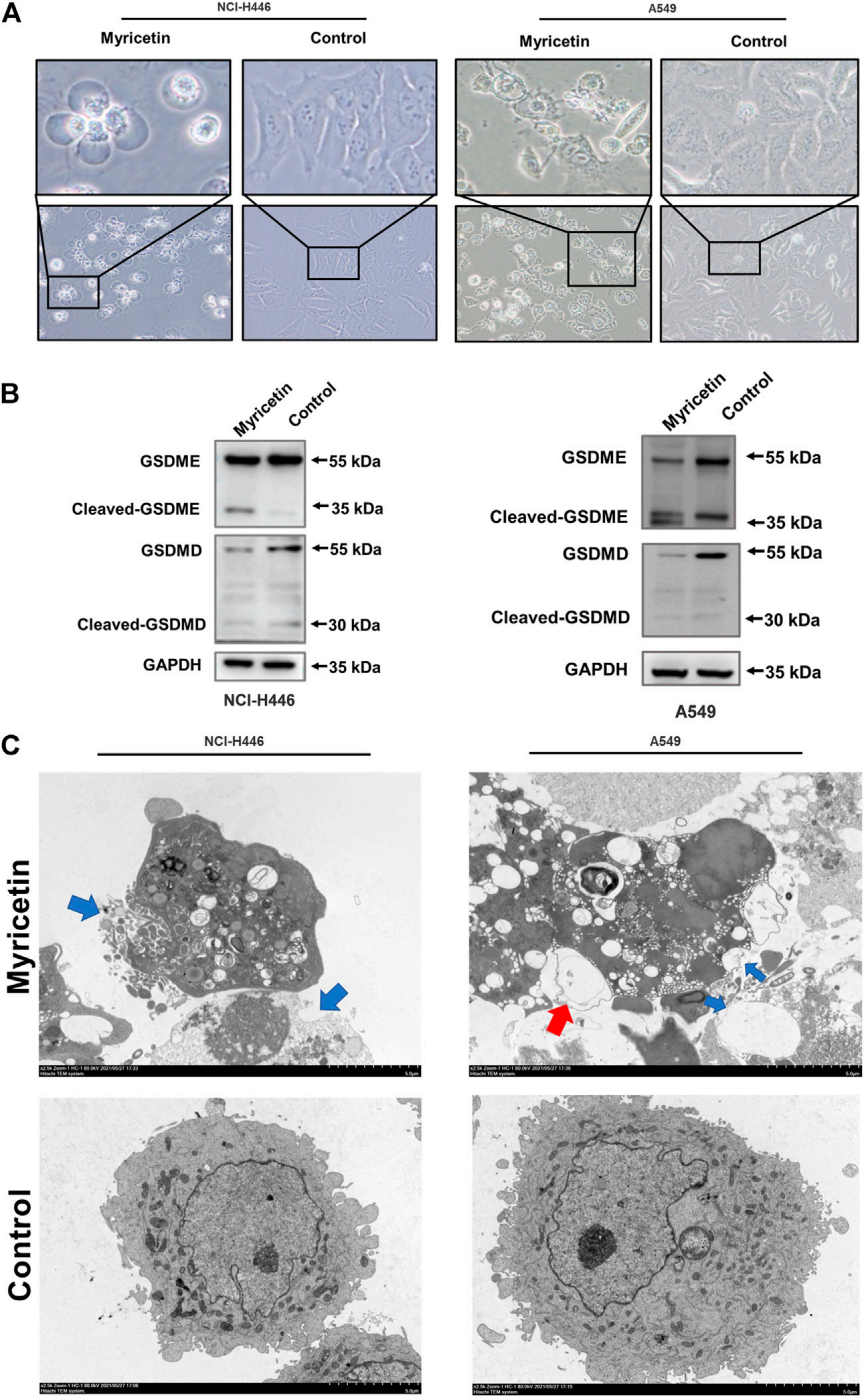
Myricetin induced pyroptosis through GSDME. **(A)** Microscopic imaging of NCI-H446 and A549 cells after myricetin (150 μM) treatment. **(B)** Expression of GSDMD and GSDME in NCI-H446 and A549 cells after myricetin (150 μM) treatment. **(C)** Transmission electron microscopy (The blue arrows refer to the holes formed in the cell membrane, and the red scissors refer to the vacuoles formed in the cell membrane). The blue arrowheads indicate the emerging pores from the plasma membranes.

**FIGURE 4:**
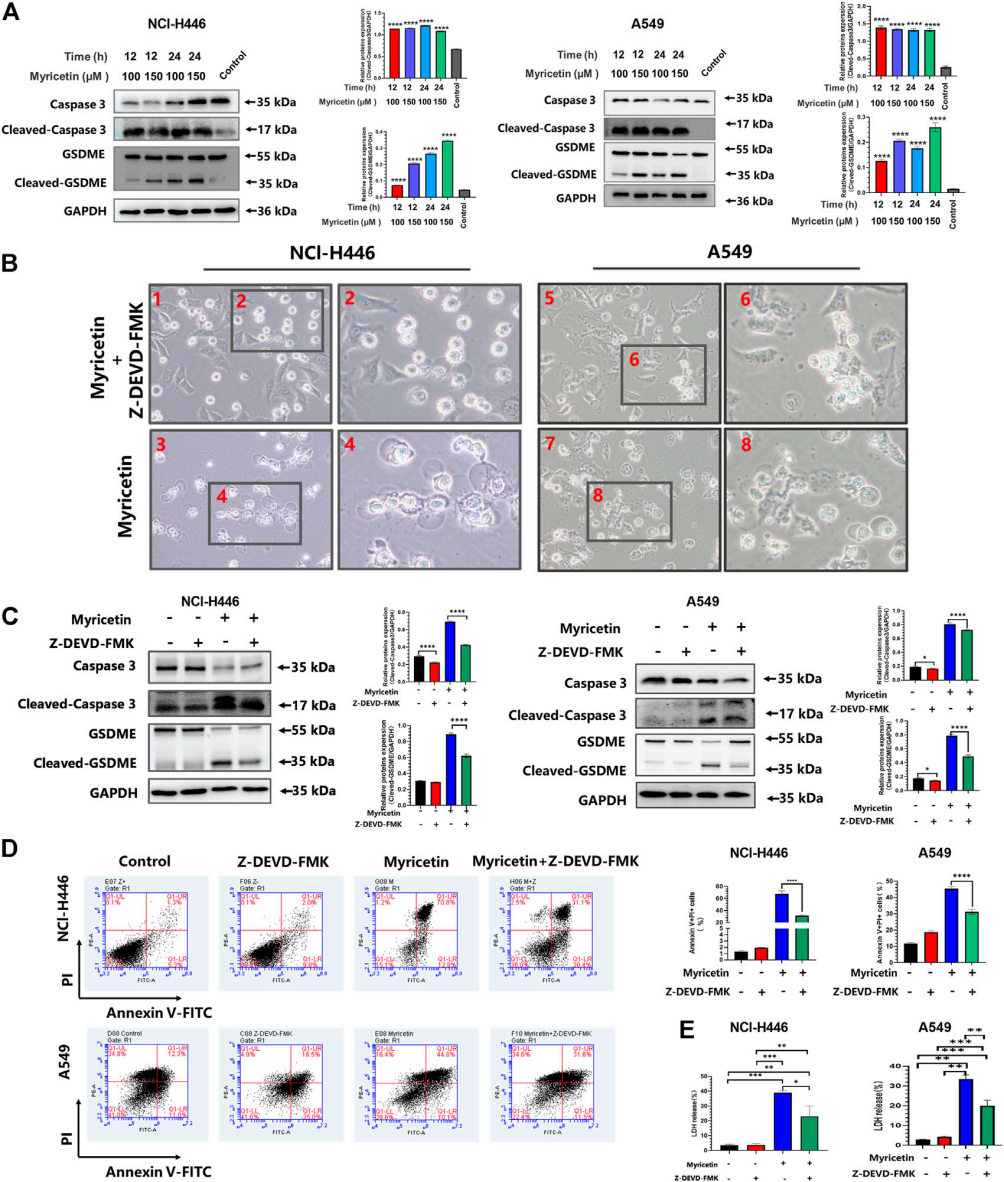
Myricetin-induced pyroptosis depends on Caspase-3. **(A)** Expression of cleaved caspase-3 and GSDME after myricetin treatment for 12 and 24 h and its statistical analysis. **(B)** Microscopic imaging of NCI-H446 and A549 cells treated with myricetin and a caspase-3 inhibitor (Z-DEVD-FMK, 20 μM) or myricetin alone. **(C)** NCI-H446 and A549 cells treated with caspase-3 inhibitor alone, myricetin alone, myricetin and caspase-3 inhibitor or mock. Expression of cleaved caspase-3 and GSDME and its statistical analysis. **(D)** Percentage of NCI-H446 and A549 cells with pyrotosis (stained with Annexin V+/PI+) and its statistical analysis. **(E)** Detection of LDH release levels. Results were presented as means ± standard deviation (*p < 0.05, **p < 0.01, ***p < 0.001, ****p < 0.0001, when compared with the control).

After treatment of NCI-H446 and A549 cells with myricetin (150 μM), the cells were observed under a microscope to detect cell swelling and empty cell membranes (Figure 3A). This morphology is obviously different from that of the classical morphology of apoptotic cells, but similar to the previously reported morphology of chemotherapy-induced pyroptosis of tumor cells. In addition, transmission electron microscopy showed the presence of many pores in the cell membranes of myricetin-treated NCI-H446 and A549 cells, which are also typical features of pyroptosis (Figure 3C). In previous studies, PI-positivity and Annexin V-positivity were always regarded as one of the indicators in the evaluation of apoptosis. Recent studies have shown that this double positive indicator can also be used to evaluate pyroptosis. As shown in Figure 4D, the number of PI-positive and Annexin V-positive myricetin-treated NCI-H446 and A549 cells was significantly higher than that of the control group (*p <* 0.0001). Therefore, we concluded that myricetin induces the cell death of lung cancer cells *via* the pyroptosis pathway. GSDMD and GSDME proteins that are the key proteins for cell scorch death, were detected by WB. The results showed that after treatment with myricetin, the change in the level of cleaved-GSDMD was not significant, but the level of cleaved-GSDME protein was significantly increased (Figure 3B). Taken together, the above results indicate that myricetin induces pyroptosis in NCI-H446 and A549 cells by cleaving GSDME instead of GSDMD.

In addition, we also analyzed the effect of myricetin on the mouse lung cancer Lewis cells and found that myricetin can also lead to their pyroptosis by activating caspase-3-mediated cleavage of GSDME (Supplementary Figure S1C). However, there were no typical morphological characteristics of pyroptosis in Lewis cells.

### Myricetin-induced pyroptosis depends on the caspase family

Different caspases are required for the occurrence and development of different cell pyroptosis, and caspase-3 is essential for GSDME pathway-dependent pyroptosis. Therefore, the activity level of caspase-3 was first detected. The results showed that myricetin increases the expression level of cleaved-caspase-3 (Figure 4A). In addition, we analyzed other pyroptosis-related caspases and found that the other caspases are not activated (Supplementary Figure S1B). Therefore, myricetin-induced pyroptosis may be dependent on the cleaved caspase-3. For further verification, we treated NCI-H446 and A549 cells with the caspase-3 inhibitor, Z-DEVD-FMK, and observed a significant reduction in pyroptosis by microscopy (Figure 4B), and an inhibition of LDH release (Figure 4E). WB results also showed that with the decrease in the expression level of cleaved-caspase-3 protein, the expression of cleaved-GSDME was also significantly decreased (Figure 4C). Therefore, we reasoned that the inhibition of caspase-3 can block the cleavage of GSDME. Meanwhile, we also used the Annexin V-FITC/PI assay on NCI-H446 and A549 cells that were treated with myricetin and the caspase-3 inhibitor, Z-DEVD-FMK, and the results were consistent with those of the WB. With the inhibition of caspase-3, the number of pyroptosis cells was significantly reduced (*p <* 0.0001) (Figure 4D). Taken together, the above results indicate that myricetin-induced pyroptosis is dependent on the caspase-3 pathway.

### Myricetin induced pyroptosis through the Ca^2+^-mediated ER stress pathway and ROS activation

**FIGURE 5:**
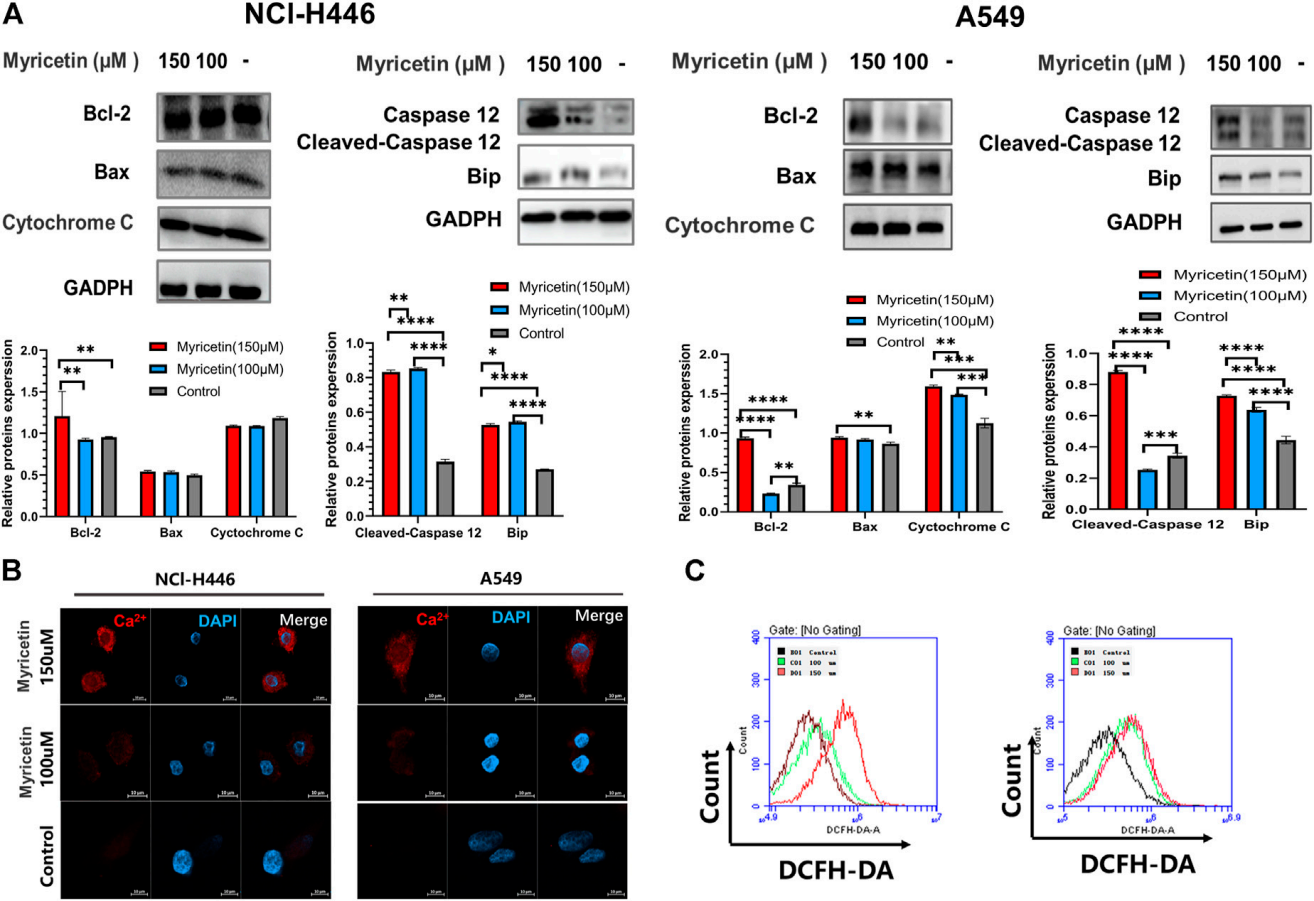
Myricetin-induced pyroptosis is associated with the endoplasmic reticulum. **(A)** Expression of mitochondrial-associated proteins (Bcl-2, Bax, and Cytochrome **(C)** after myricetin treatment and their corresponding statistical analyses. Expression of ER-associated proteins (Caspase-12 and Bip) after myricetin treatment and its statistical analysis. **(B)** Laser confocal microscope image of Ca^2+^staining after NCI-H446 cells treated with myricetin. **(C)** Analysis of ROS after NCI-H446 cells treated with myricetin. The results are presented as mean ± standard deviation (**p < 0.01, ***p < 0.001, ****p < 0.0001).

Studies have pointed out that the occurrence of cell pyroptosis is regulated by mitochondria and the endoplasmic reticulum (Zeng and Xie, 2009; Liu et al., 2022). Therefore, we also analyzed whether the mitochondria or endoplasmic reticulum (ER) is involved in myricetin-induced cell pyroptosis. For this, we detected the changes in the levels of mitochondria and endoplasmic reticulum related proteins in myricetin-treated NCI-H446 and A549 cells treated using WB (Figure 5A). The results showed that the level of expression of mitochondrial related proteins does not significantly change, but the levels of the ER related proteins, caspase-12 and BIP, are significantly changed. However, ROS detection showed that myricetin can significantly increase the level of ROS in cells (Figure 5C). In addition, we also performed a Ca^2+^ staining of myricetin-treated NCI-H446 and A549 cells, and the results showed that Ca^2+^ release increases with the increase in myricetin concentration (Figure 5B). Therefore, we deduced that myricetin-induced cell pyroptosis may be caused by Ca^2+^ induced ER stress.

**FIGURE 6:**
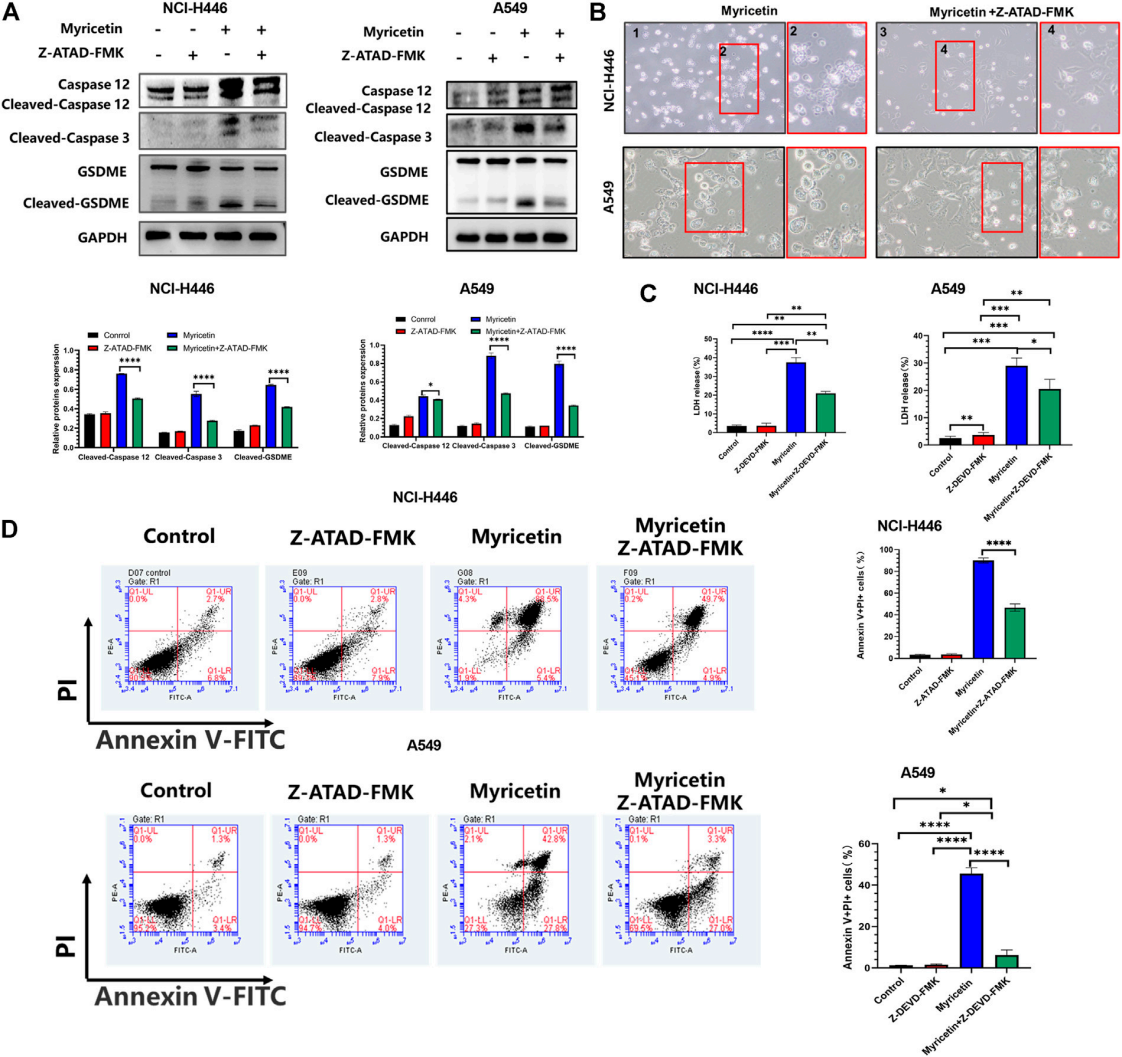
Myricetin induced pyroptosis through the ER Stress pathway. **(A)** NCI-H446 and A549 cells treated with caspase-12 inhibitor alone, myricetin alone, myricetin and a caspase-12 inhibitor or mock. Expression of cleaved caspase-12, caspase-3, and GSDME and its corresponding statistical analysis. **(B)** Microscope images of NCI-H446 and A549 cells treated with myricetin and a caspase-12 inhibitor (Z-ATAD-FMK, 50 μM) or myricetin alone. **(C)** Detection of LDH release levels. **(D)** Percentage of NCI-H446 and A549 cells with pyrotosis (stained with AnnexinV+/PI+) and its statistical analysis. Results were presented as mean ± standard deviation (**p < 0.01, ***p < 0.001, ****p < 0.0001).

For further validation, we treated NCI-H446 and A549 cells with the caspase-12 inhibitor, Z-ATAD-FMK, and observed a significant reduction in cell pyroptosis by microscopy (Figure 6B). WB results also showed that with the decrease of cleaved-caspase-12 protein expression, the expressions of cleaved-caspase-3 and cleaved-GSDMDE proteins were significantly decreased significantly after the addition of caspase12 inhibitor (Figure 6A). LDH release was also inhibited (Figure 6C). Therefore, we deduced that the inhibition of caspase-12 can block the cleavage of GSDME. Meanwhile, we also used the Annexin V-FITC/PI assay following treatment of NCI-H446 and A549 cells with myricetin and the caspase-12 inhibitor, Z-ATAD-FMK, and the results were consistent with those of the WB. With the inhibition of caspase-12, the number of pyroptosis cells was significantly reduced (*p <* 0.0001) (Figure 6D). Based on the above results, we confirmed that myricetin -induced pyroptosis was caused by Ca^2+^ induced ER stress.

### Inhibitory effect of myricetin and induced pyroptosis *in vivo*

**FIGURE 7:**
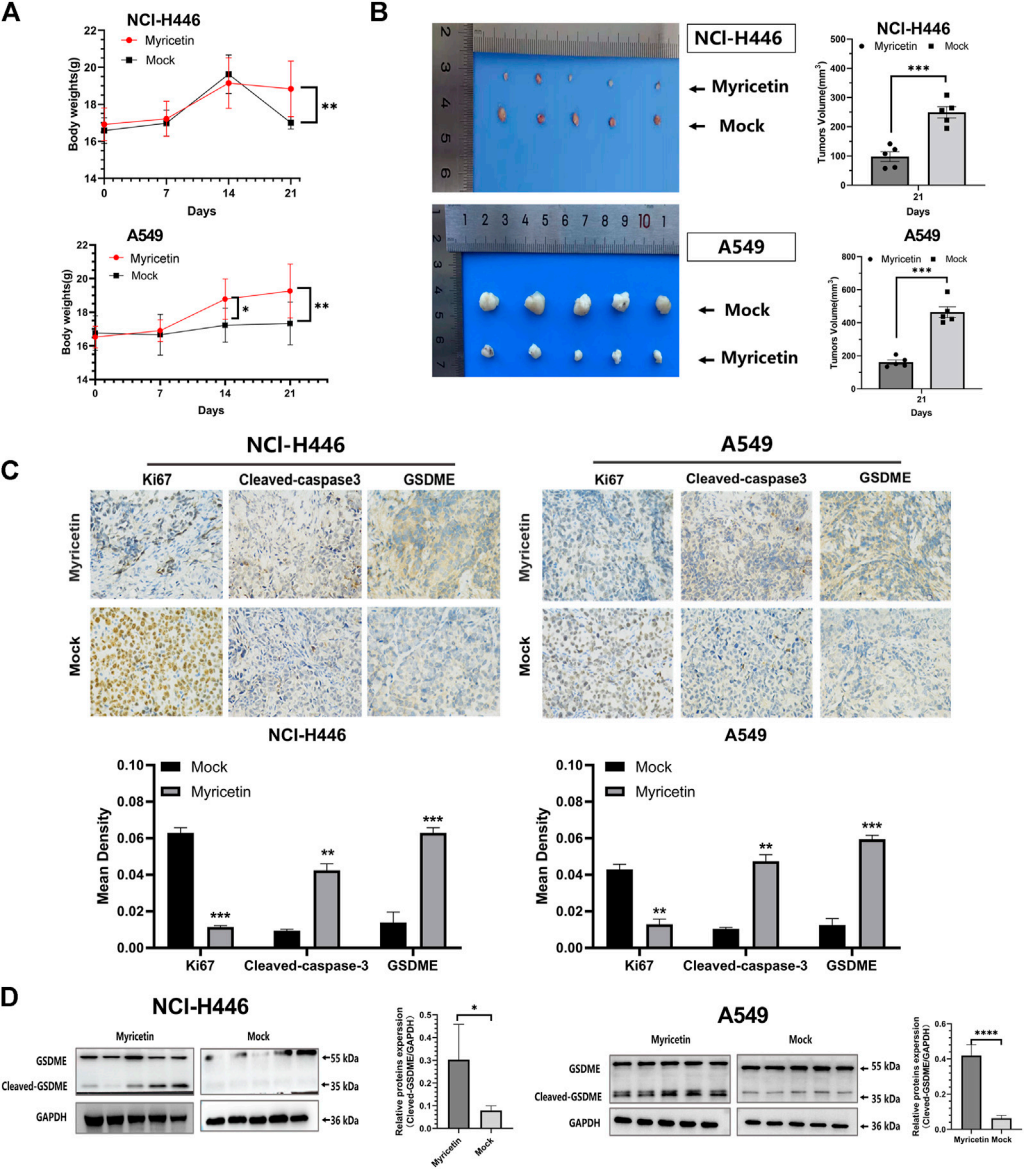
The antitumor effect of myricetin *in vivo*. Myricetin was intratumorally injected to the mice every 2 days for 2 weeks. Body weight **(A)**, images of xenografted tumors **(B)**, and tumor volumes **(B)** were recorded. **(C)** Analysis of Ki67 and immunohistochemistry (cleaved-caspase-3 and GSDME) results from tumor samples. **(D)** Western blot analyses of GSDME from tumor samples and its corresponding statistical analysis. The results were presented as mean ± standard deviation (**p < 0.01, ***p < 0.001, when compared with the Mock).

To explore the inhibitory effect of myricetin on tumor cells *in vivo*, NCI-H446 and A549 cells were subcutaneously injected into nude mice and treated with myricetin. At day 21, the weight of mice in the untreated group was reduced compared with that of the Myricetin-treated group (Figure 7A). After the nude mice were euthanized, the tumors were photographed and their tumor size was measured (Figure 7B). The results showed that the tumors’ size in the myricetin treatment group was significantly smaller than that of the control group, indicating that myricetin can effectively inhibit lung cancer cells *in vivo*. Tumor tissues were used for immunohistochemical analysis of Ki67, the proteins of GSDME, and Cleaved-Caspase 3 (Figure 7C). The results of Ki67 immunohistochemistry showed that myricetin can effectively inhibit the proliferation of tumors. The immunohistochemical results also showed that GSDME and Cleaved-Caspase 3 were significantly higher than those in the untreated group. For further verification, WB was performed on protein extracts from tumor tissues, and the results showed that the tumors treated with myricetin expressed cleaved-GSDME proteins (Figure 7D). In summary, myricetin can inhibit tumor growth and induce cell pyroptosis *in vivo*.

### Model of myricetin activation of caspase-3/GSDME induced pyroptosis in lung cancer

**FIGURE 8:**
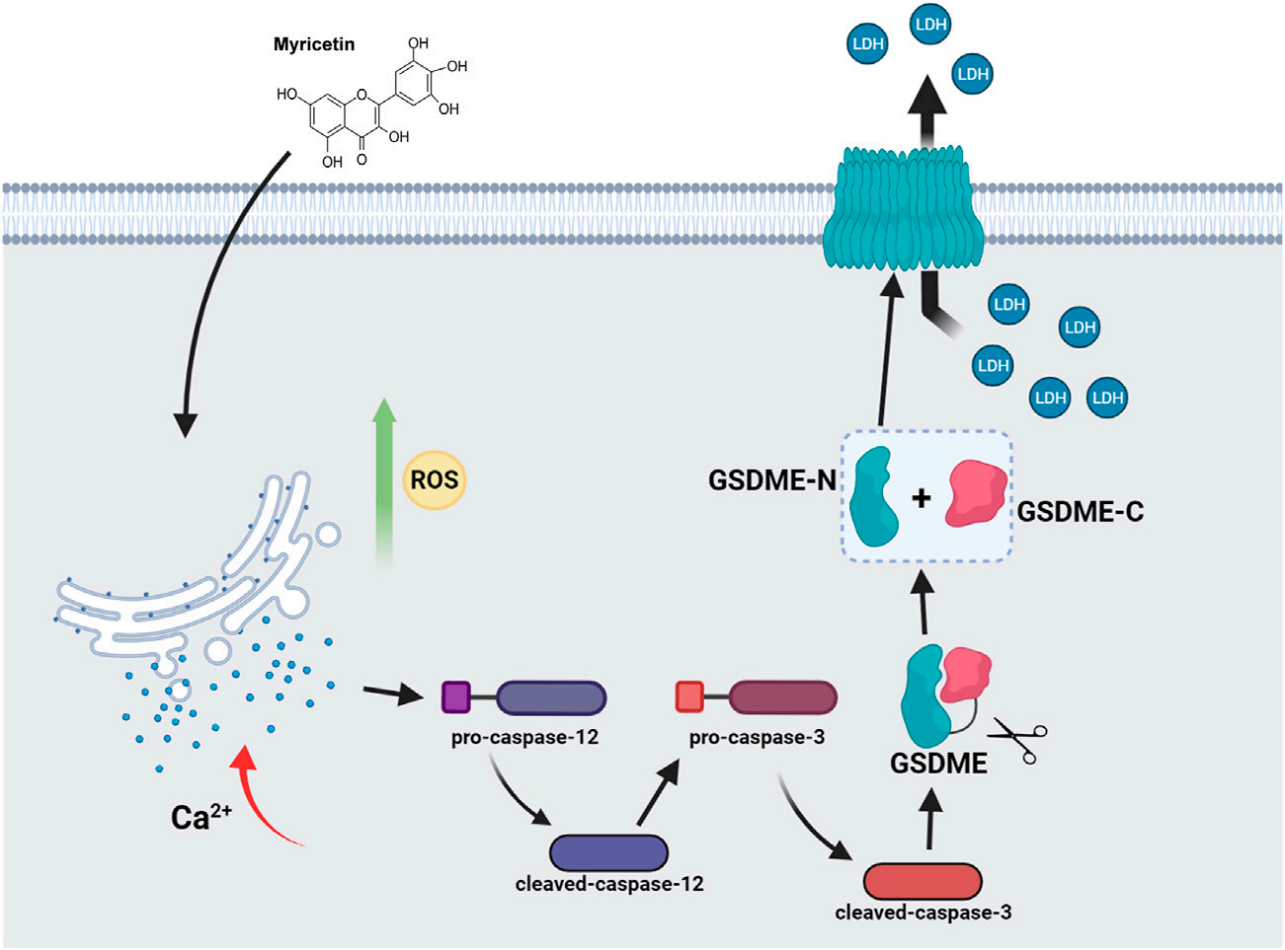
Model of myricetin activation of caspase-3/GSDME induced pyroptosis in lung cancer.

Myricetin can activate ROS and ER stress and release Ca^2+^ into the cytoplasm through the ER stress pathway. ER stress then activates caspase-12 and subsequently caspase-3. GSDME is cleaved by cleaved-caspase-3 to generate GSDME-N, where GSDME-N can directly oligomerize, release LDH, and cause plasma membrane lysis, leading to lung cancer pyroptosis (Figure 8).

## Discussion

Lung cancer has the highest mortality rate and the second incidence rate among all cancers. Clinically, lung cancer can generally be divided into non-small cell lung cancer (NSCLC) and small cell lung cancer (SCLC) (Li Y. et al., 2022). The two lung cancer subtypes significantly differ in histological type, biological behavior, incidence, prognosis, and treatment response (Siegel et al., 2021; Li Y. et al., 2022). As lung cancer is complex and refractory, more researchers are focusing on adapting the use of existing drugs in cancer treatment. One approach is to explore the anticancer potential of existing drugs, and reduce the time and cost of research by acquiring large amounts of data, thereby increasing success rates. The second way is to expand the indications of the drug. Many lung cancer patients are at an advanced stage of cancer that requires systemic treatment. Despite the high response rate of platinum/etoposide combination therapy, the median overall survival (OS) is only about 10 months (Gadgeel, 2018). Therefore, there is still a need to develop new drugs for the treatment of lung cancer patients.

Myricetin is a natural flavonol compound that exists in a variety of plants, including berries, oranges, grapes, herbs, wines, and many types of teas (Jiang et al., 2019). In the past decades, many studies the various biological activities of myricetin, such as antiviral, anti-inflammatory, antioxidant, and anti-tumoral activities. For example, myricetin is used in the treatment of diseases such as infectious bronchitis virus (IBV), osteoporosis, diabetes, atherosclerosis, and vascular restenosis. Myricetin is also used in research and development of anti-tumor drugs, such as in the treatment of breast, colon, liver, gastric, bladder, skin, prostate, and thyroid cancers. However, myricetin has different effects on different cancer cells. For example, myricetin induces apoptosis and regulates JNK-mediated autophagy in SK-BR-3 human breast cancer cells through the MAPK pathway, while it induces cellular oxidative stress and produces intracellular ROS through the Fenton reaction in triple negative breast cancer cells (Knickle et al., 2018). Myricetin induces treatment-related apoptosis and autophagy in colon cancer by inhibiting the PI3K/Akt/mTOR signaling pathway (Zhu et al., 2020). In the treatment of prostate cancer, myricetin promotes prostate cancer cell apoptosis and antimetastatic effects by inhibiting PIM1 and by disrupting its interaction with CXCR4 (Ye et al., 2018). In gastric cancer, myricetin can bind to RSK2, resulting in an increased expression of Mad1, and enhancement of the inhibition of HGC-27 and SGC7901 cells’ proliferation (Feng et al., 2015).

The above research on the effect of myricetin on tumor cells shows that myricetin can inhibit many types of tumors, but its mechanism is different from one type of tumor to another. Currently, there is little research on the effect of myricetin on lung cancer cells, and therefore, this study explored the inhibitory effect of myricetin on lung cancer cells. First, we found that myricetin can effectively inhibits the migration and invasion of lung cancer cells (Figures 2A,B). E-cadherin is considered as an inhibitor of tumor migration and invasion through maintaining an epithelial phenotype, while Snail is an important promoting factor of epithelial-mesenchymal transition (EMT), which plays a key role in the EMT pathway associated with tumor invasion and metastasis (Chatterjee et al., 2017; D'Alterio et al., 2020; Giannelli et al., 2016). Therefore, we detected the expression of E-cadherin and Snail proteins by Western Blot. Following the treatment of lung cancer cells with myricetin and found that the expression level of E-cadherin significantly increases and that of Snail significantly decreases (Figure 2C). Then we found that myricetin can effectively inhibit lung cancer cells using various *in vitro* experiments. This inhibitory effect was significantly higher than that of the control group (Figures 1B,C). However, the mechanisms of these inhibitory effects are unclear, and therefore, we explored these mechanisms in this study. Following myricetin treatment of lung cancer cells, we observed the cells under a light microscope and a transmission electron microscope and found that lung cancer cells were swollen and with the presence of many bubble-like protrusions on the surface of the cell membranes (Figure 3A). Transmission electron microscope analysis showed the presence of non-specific holes on the surface of lung cancer cells, and damages to the integrity of the cell membranes, which led to an overflow of cell contents (Figure 3C). After treatment with myricetin, lung cancer cells showed typical cell pyroptosis characteristics.

Pyroptosis is defined as a programmed cell death mediated by the gasdermin family of proteins. At present, among these proteins, GSDMD and GSDME have been thoroughly studied for their role in cell apoptosis (Hu et al., 2020). GSDMD-induced apoptosis is generally mediated through a caspase-1-dependent classical pathway and a caspase-4/5/11-dependent nonclassical pathway. GSDMD is activated by caspase-1 or caspase-11/4/5, and its N-terminal domain can oligomerize to form cell membrane pores, that induce cell membranes’ rupture (Yu et al., 2021). On the other hand, GSDME can induce pyroptosis by activating caspase-3 to cleave GSDME following treatment with chemotherapeutic agents (Wang et al., 2017). In this study, we analyzed GSDMD and GSDME protein expression levels after treatment of lung cancer cells with myricetin. The results showed that GSDME is cleaved and produces a GSDME-N fragment (Figure 3B). Therefore, we hypothesized that myricetin induces cell pyroptosis by cleaving GSDME.

Existing studies have shown that chemotherapeutic drugs can induce caspase-3-mediated cleavage of GSDME, resulting in high GSDME expression and the formation of N-GSDME terminals, which lead to tumor cell pyroptosis (Wang et al., 2017). Thus, we explored the role of caspase-3 in the pyroptosis of lung cancer cells following treatment with myricetin. We found that cleaved-caspase-3 and cleaved-GSDME are highly expressed (Figure 4A). To further verify the effect of caspase-3, we treated the cells with the caspase-3 inhibitor and found that the number of cells exhibiting pyroptotic characteristics is significantly reduced (Figure 4B). In addition, the addition of Z-DEVD-FMK significantly reduced the cleavage of GSDME (Figure 4C), the release of LDH (Figure 4E), and the proportion of AnnexinV+/PI + positive cells (Figure 4D). Taken together, the above results demonstrate that myricetin can mediate the cleavage of GSDME by activating caspase-3, resulting in pyroptosis. In addition, we also analyzed the effect of myricetin on the mouse lung cancer Lewis cells and found that myricetin can also lead to pyroptosis by activating caspase-3-mediated cleavage of GSDME (Supplementary Figure S1C).

Many studies have pointed out that the occurrence of cell pyroptosis is regulated by mitochondria and the endoplasmic reticulum (ER) (Zeng and Xie, 2009; Yu et al., 2021; Liu et al., 2022). Therefore, we investigated whether these organelles are involved in the induction of cell pyroptosis by myricetin. For this, we analyzed the changes in the levels of mitochondria and ER-related proteins after treatment of lung cancer cells with myricetin. The results showed that the levels of expression of the ER-related proteins, Caspase-12 and BIP, were significantly changed (Figure 5A). In addition, Ca^2+^ staining results showed that the release of Ca^2+^ increases with the increase of myricetin concentration (Figure 5B). Therefore, we deduced that myricetin-induced cell pyroptosis may be caused by Ca^2+^-induced ER stress. To further verify the relationship between cell pyroptosis and ER stress, we added inhibitors of key proteins of ER stress to clarify whether ER stress was involved in caspase-3-mediated cleavage of GSDME leading to cell pyroptosis. ER stress is a reaction process in which cells activate signaling pathways such as protein unfolding response, endoplasmic reticulum overload response, caspase-12-mediated apoptosis pathway in response to misfolded and unfolded protein aggregation in the ER cavity, dysregulation of calcium homeostasis, and other conditions (Gorman et al., 2012). Besides, all discovered pathways for caspase-12 activation are related to ER stress. The activation of caspase-12 eventually leads to the activation of downstream executive caspases (such as caspase-3,6,7) and cell death (Xu et al., 2019). In this study, we found that myricetin had no activating effect on caspase-6 and 7, but only caused the activation of caspase-3 (Supplementary Figure S1B). After adding the caspase12 inhibitor, Z-ATAD-FMK, pyroptosis was significantly reduced when observed by microscopy (Figure 6B). Moreover, the addition of Z-ATAD-FMK significantly reduced the cleavage of caspase-3 and GSDME (Figure 6A), LDH release (Figure 6C), and the Annexin V+/PI + positive cell ratio (Figure 6D). Taken together, the above results confirm that myricetin relies on ER stress to induce pyroptosis of lung cancer cells.

ROS are closely related to tumor cell death. Excess of ROS enhances cellular oxidative stress, resulting in DNA, protein, or lipid damage, that leads to cell death. Therefore, increasing the level of ROS in tumor cells by chemotherapeutic drugs has been applied in clinical cancer treatment. In this study, we found that myricetin cannot cause changes in mitochondria-related proteins and the cells ROS level was also significantly increased with the increase in drug dose, indicating that myricetin may also cause the death of lung cancer cells by activating ROS (Figure 5C).

To further clarify the effect of myricetin on lung cancer, we performed an *in vivo* study. The results showed that myricetin can effectively slow down the effect of tumors on mice weight (Figure 7A) and significantly inhibit the proliferation of lung cancer cells *in vivo* (Figure 7B). Immunohistochemistry and WB using tumor tissues showed that the results were consistent with those of the *in vitro* studies. Myricetin could promote the high expression of cleaved-GSDME in tumor tissues (Figures 7C,D). In summary, myricetin can also inhibit tumor growth and induce cell pyroptosis *in vivo*.

The molecular mechanisms of pyroptosis include the classical pathway mediated by caspase-1, the non-canonical pathway mediated by caspase-4/5/11, the pathway mediated by caspase-3, and the granzyme-mediated pathway, among which the caspase-3 pathway mainly induces pyroptosis by cleaving GSDME (Yu et al., 2021). In this study, we found that myricetin induces pyroptosis in lung cancer cells through endoplasmic reticulum stress that leads to the activation of caspase-3 and the cleavage of GSDME. However, this study has some limitations. For instance, caspase-3 and caspase-12 inhibitors were added to inhibit caspase-3 and caspase-12, and the results showed that the addition of the inhibitors significantly reduces the cleavage of GSDME protein and the changes of pyroptosis-related indicators, which was consistent with the previous research results of GSDME pathway-induced pyroptosis (Zhang et al., 2020). It also proved that myricetin activates caspase-3 and cleaves GSDME to induce pyroptosis in lung cancer cells. In this study, we only verified the pyroptosis induced by the GSDME pathway by adding inhibitors but did not further verify the effect of complete knockout of caspase-3 and caspase-12 on GSDME. Therefore, we will further improve the study of pyroptosis in future studies.

## Conclusion

In conclusion, the inhibitory effect of myricetin on lung cancer cells was explored *in vivo* and *in vitro*, and we confirmed that myricetin has a significant inhibitory effect on lung cancer. In addition, a new mechanism of myricetin-mediated cell death of tumor cells was discovered. This mechanism involves myricetin activation of caspase-3 through the ER stress pathway to promote the cleavage of GSDME and trigger cell pyroptosis. This is significantly different from what was previously reported with other tumor cells which indicate that antitumor effects are mediated by the apoptotic pathway. By revealing this new anti-tumor mechanism, the study provides a new theoretical basis for the development of anti-lung cancer drugs.

## Data Availability

The original contributions presented in the study are included in the article/Supplementary Material, further inquiries can be directed to the corresponding authors.

## Supplementary material

Supplementary Figure S1
**(A)** Half maximal inhibitory concentration (IC50) of Myricetin used for the inhibition of the proliferation of BEAS-2B cells. **(B)** Western blot analyses of the expression of cleaved caspase-3/4/5/6/7/8 in NCI-H446 cells after myricetin treatment. **(C)** Myricetin induced pyroptosis in Lewis cell by GSDME. **(D)** Microscopic imaging of Lewis cells after myricetin treatment.

## Abbreviations

DC, dendritic cells; ER, Endoplasmic reticulum; GSDM, gasdermin (GSDM); NSCLC, non-small cell lung cancer; PCD, programmed cell death; PJVK, Pejvakin; SCLC, small cell lung cancer.
