# Supplementary figures and images for: Myricetin activates the Caspase-3/GSDME pathway via ER stress induction of pyroptosis in lung cancer cells
Source: Front Pharmacol. 2022 Aug 26;13:959938. doi: 10.3389/fphar.2022.959938 (PMC9458876; doi:10.3389/fphar.2022.959938)

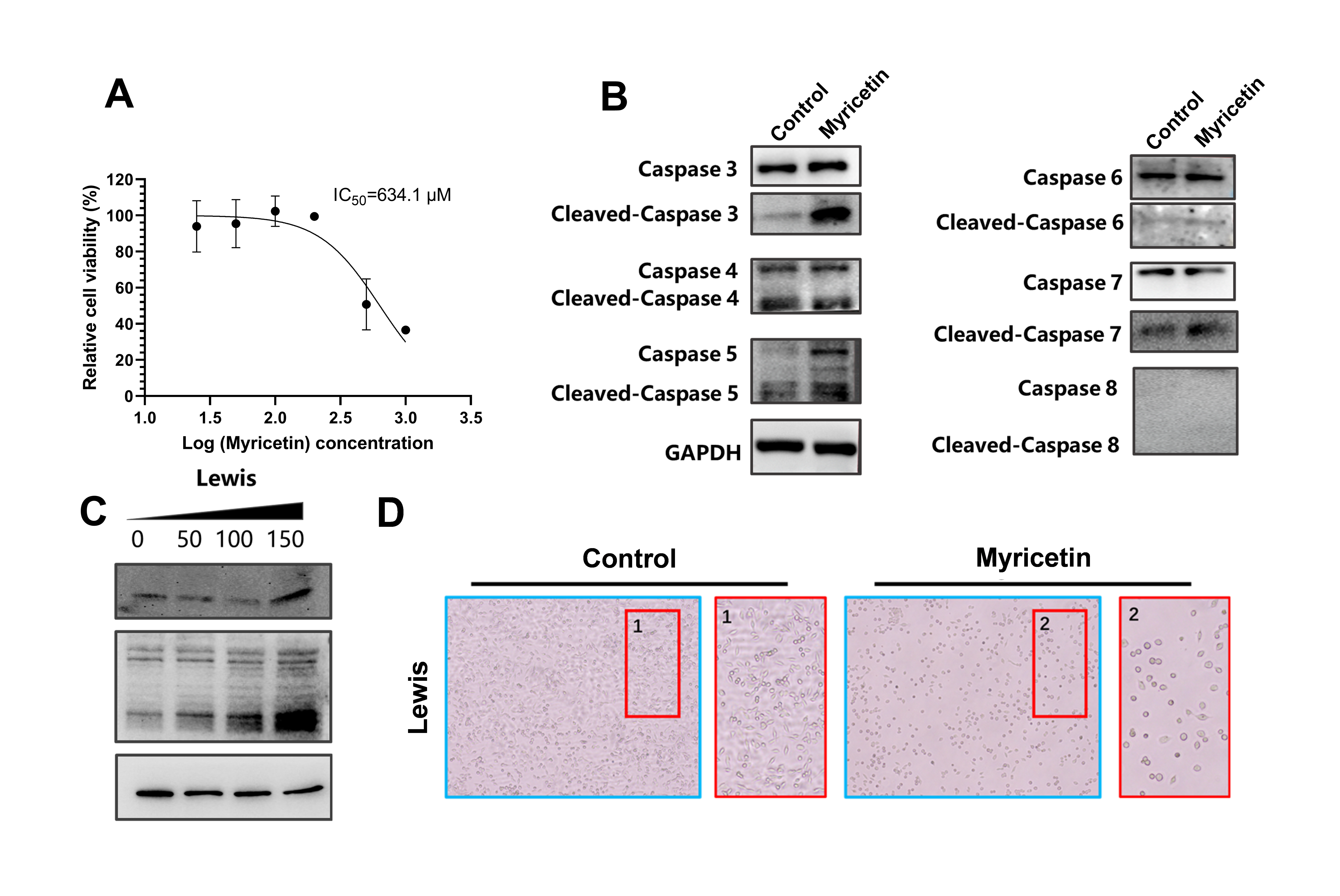

Supplement: Supplementary file 1 [file Image1.tif]
